# Electroanalytical Trace Metal Cations Quantification and Speciation in Freshwaters: Historical Overview, Critical Review of the Last Five Years and Road Map for Developing Dynamic Speciation Field Measurements

**DOI:** 10.3390/molecules28062831

**Published:** 2023-03-21

**Authors:** José Paulo Pinheiro, Elise Rotureau

**Affiliations:** Université de Lorraine, CNRS, Laboratoire Interdisciplinaire des Environnements Continentaux (LIEC), F-54000 Nancy, France

**Keywords:** field measurements, electroanalytical techniques, trace metals, equilibrium speciation, dynamic speciation, SCP, SSCP, AGNES

## Abstract

An historical overview covering the field of electroanalytical metal cations speciation in freshwaters is presented here, detailing both the notable experimental and theoretical developments. Then, a critical review of the progress in the last five years is given, underlining in particular the improvements in electrochemical setups and methodologies dedicated to field surveys. Given these recent achievements, a road map to carry out on-site dynamic metal speciation measurements is then proposed, and the key future developments are discussed. This review shows that electroanalytical stripping techniques provide a unique framework for quantitatively assessing metals at trace levels while offering access to both thermodynamic and dynamic features of metal complexation with natural colloidal and particulate ligands.

## 1. Introduction

Metal ions in natural waters interact with a wide assortment of dissolved inorganic and organic ligands, adsorb onto natural colloids, suspended matter and at micro-organism surfaces (where they can be possibly internalized). In addition to their large polydispersity in size, most of these natural complexing ligands, surfaces or particles present a polyfunctional and polyelectrolytic character, thus displaying a large range of complex formation/dissociation rate constant, hence controlling the bioavailability and mobility of the metal ions [1].

Natural waters are systems that typically never reach a state of chemical equilibrium; therefore, a correct diagnostic of the fate and environmental impact of metal complexes must consider the importance of both the reactivity and mass transport fluxes of the metal compounds and the relative time scales of these processes (dynamic speciation) [2]. Concerning the physico-chemical theoretical description of the metal speciation, it is interesting to observe that, albeit the knowledge of dynamic trace metal cation speciation is the key to understanding the mobility, bioavailability and toxicity in freshwaters, the most common theoretical approaches to describe these phenomena are still thermodynamic in nature. In this review, a brief historical overview of the development of thermodynamic and dynamic theories will be provided.

The most frequent analysis carried out in freshwater samples is the quantification of total metal concentration, which, in practice, consists of strongly acidifying the sample, coupled or not with the destruction of the natural organic matter (NOM), and sending it to a laboratory for quantification by means of an Inductively Coupled Plasma-Mass Spectroscopy (ICP-MS) [3]. To understand trace cation bioavailability or toxicity, the total metal concentration determination is clearly not sufficient, and it is necessary to evaluate at least the free metal ion and the different metal complexes concentrations. From an experimental point of view, freshwater samples are very complex media; therefore, a simplification is made to reduce the polydispersity of the sample and protect sensitive analytical instruments, i.e., it is conventionally adopted that the samples are filtered using generally 0.2 or 0.45 (to a lesser extend now) µm pore-sized filter, yielding a so-called “dissolved fraction” and collecting larger particles and microorganisms in the filter [4], even though Nakai [5] warned that many micro-sized bacteria may likely pass through a 0.2 μm membrane.

In the “dissolved” fraction, trace metal cations are predominantly bound either by NOM, which is an ensemble of negatively charged, chemically heterogeneous and polydisperse small organic colloids [6], or by small inorganic colloids such as iron or manganese oxides or clay minerals [7,8,9,10]. We recall here the IUPAC definition of a colloid as being “molecules or polymolecular particles dispersed in a medium that have at least in one direction a dimension roughly between 1 nm and 1 µm” [11]. Hence, the free hydrated cation is usually a small fraction of the total metal present in solution.

One of the major problems is that sampling and handling of natural water samples can introduce changes in the metal species distribution prior to measurement [12]. Therefore, it is preferable to carry out field measurements to avoid contamination during sampling and storage, metal adsorption to container walls, and changes in speciation through processes associated with aggregation, re-equilibration of gaseous components and precipitation during transport and storage prior to measurement. Over the years, extensive effort has been made to design chemical sensors for in situ and on-site measurements in aquatic environments, including continuous flow analyzers as well as optical and electrochemical sensors [13].

In the last three decades, trace metal cations field measurements have been performed using two approaches: (i) the deployment of in situ exposure devices which accumulate metal (free and dynamic metal species) such as the Diffusion Gradient in Thin Films (DGT) [14] or the Permeation Liquid Membranes (PLM) [15], or equilibrated with free metal ions only, i.e., Donnan membrane Technique (DMT) [16], or (ii) electroanalytical devices such as the Gel Integrated Mercury Electrode (GIME) [17], stripping voltammetric techniques (SV) (Chapter 5 in [18]) and competitive ligand exchange/cathodic stripping voltammetry (CLE-CSV) [19,20].

DGT techniques have gained significant preponderance for in situ exposure due to being very easy to use; nonetheless, the interpretation of the signal is quite involved [21]. The gel barrier is not really effective since large particles can penetrate the gel phase [22], and humic matter can accumulate at the gel/solution interphase [23], further complicating DGT data interpretation [24]. In theory, PLM should be able to obtain free (with thick membrane configuration) or dynamic metal speciation information (with thin membrane configuration) [25], even so, in practice, it was found that in the later configuration, it presented larger measurement uncertainty that prevented the computation of reliable dynamic information [26]. DMT is a robust technique that provides reliable quantification of the free hydrated cation in multicomponent solutions [27] and natural waters [28]. Nevertheless, its ability to provide metal speciation dynamic information is very limited [29].

In this review, the focus will be on the stripping electroanalytical techniques for their unique ability to perform equilibrium and dynamic speciation at very low quantification limits, low cost, and portability. Stripping techniques are carried out in two steps: in the first step (deposition step), a fixed potential (deposition potential) is applied between the working and reference electrodes, leading to the reduction in the metal ion of interest, which becomes amalgamated at the mercury working electrode or deposited at the surface of a solid working electrode. In the second step (reoxidation step), the deposited metal is re-oxidized in a controlled way in order to quantify exactly the amount deposited during the first step [30].

Development of field electroanalytical techniques has been led by the seawater community due to the success of CLE-CSV in measuring extremely low concentrations of metals ions (especially iron). This is because the instrumentation can be easily operated in a ship, and the quality of electroanalytical measurements is improved in very high salinity and naturally pH-buffered solutions. It is worth mentioning that on-field studies using CLE-CSV has been published for freshwaters systems to detect a wide variety of metallic elements such as Pb [31], Se [32] Cu and Zn [33], Co [34], Cd [35] or Ni [36]. In this report, we opt to only cover speciation methodologies dedicated to freshwater and will only address estuarine or seawater studies for especially meaningful developments. Readers interested in speciation in the marine or estuarine environment can consult two recent reviews in this subject by Laglera and Monticelli [37] and Cuartero [38].

In summary, a complete trace metal cation speciation analysis comprises the quantification of total metal, free hydrated cation and the different complexed and adsorbed species (mineral particles, microorganisms) with additional insight into the dynamic nature of said complexes. To achieve this goal, one needs first to be able to measure trace metal cation speciation on-site with minimal interference with the matrix, and secondly, to have a proper thermodynamic and kinetic physico-chemical description of the trace metal speciation to interpret the measures correctly.

The objective of this document is, therefore, to offer a critical review of both the electroanalytical field measurements (and associated methodological developments) and the advances on the thermodynamic and kinetic theoretical frameworks that describe trace metal speciation in natural waters and to provide a road map to develop field trace metal cation dynamic speciation.

## 2. Critical Review

### 2.1. Theoretical Background

Studying the dynamic speciation of trace metal cations is a key aspect to a better assessment of bioavailability and toxicity of metal complexes, that is, whether or not they can contribute according to the dynamic features of their binding properties, to the amount of metal internalized by a living organism. Nonetheless, since the development of the Free Ion Activity Model (FIAM) in the 1970s [39] and its direct successor, the Biotic Ligand Model (BLM), in the early 2000s [40], only the free metal ion was considered toxic by the ecotoxological community. Therefore, the interest in trace metal speciation was strongly biased towards the thermodynamic (equilibrium) aspects, focusing on the estimation of free metal activity, especially its interaction with NOM.

Historically, soil scientists carried out pioneering studies on metal cation binding with NOM. Soil is a solid and complex matrix, which makes it practically impossible to directly examine metal ion interaction with soil organic matter. To carry out these studies, soil organic matter was extracted and operationally separated into different fractions according to their solubility, yielding three components: fully soluble fulvic acids (FA), humic acids (HA) that precipitated in acidic conditions, and finally, the insoluble fraction named humin. By analogy with the soil procedure, NOM from aquatic systems is isolated using an XAD-8 column (hydrophobic fraction) and separated in FA and HA by acid precipitation of the later [41].

Understanding the reactivity of the charged humic substances toward aqueous metal cations requires a correct description of both electrostatic and chemical contributions. Currently, proton and metal cation binding to FA/HA is performed using one of the two leading semi-empirical models NICA (Non-Ideal Competitive Adsorption) [42] and WHAM (Windermere Humic Aqueous Model) [43], both relying on a ‘Donnan-like’ model to describe the effects of colloidal charge on the non-specific electrostatic metal binding to particle body. However, the application of the Donnan model for humic substances has often been criticized in the literature for its inappropriate description of the electrostatic characteristics, especially when the NOM colloids are nanoparticles-sized [44]. The Donnan model is only valid for colloids with a radius that is much larger than the thickness of the intraparticulate Debye layer and that possess a sufficiently high charge density so that the electrical potential can be considered homogeneous within the particle volume [45]. Nonetheless, in freshwater, a considerable portion of the NOM is constituted by very small colloids, for example, fulvic and small humic acids, that do not meet these criteria.

Albeit the approximations used in these models, their development has almost stopped during the last two decades after the publication of ‘’generic’’ parameters for NICA-Donnan for both proton and metal ions [46] in the early 2000s and the implementation of WHAM (with generic parameters) in the free geochemical program “PhreeqC”.

Only recently, a revival of the speciation computation was observed in the literature, first by Janot et al. [47], who presented the coupling NICA-Donnan model with the free parameter estimation code “PEST”, followed by the replacement of the Donnan model by a more physico-chemically robust electrostatic model based on the Poisson-Boltzmann theory (Soft Poisson Boltzmann Titration—SPBT) proposed in 2021 by Pinheiro et al. [48]. Additionally, Tesfa et al. [49] in 2022 presented a modified protolytic titration methodology that is significantly faster (two days instead of one week) and produces more reliable proton titrations datasets, thus yielding better proton binding parameters upon fitting using SPBT/PEST.

It is necessary to remind the reader that these thermodynamic descriptions are approximations since metal cations in freshwaters are dynamic entities that never really reach equilibrium. Nonetheless, for over two decades, between 1970 and 1990, the free ion activity model was the paradigm in the domain of trace metal cation toxicity. This status quo began to be attacked in the marine literature in the 1990s following on the work of Morel and Hudson [50,51,52], who suggested kinetic and thermodynamic controls on metal uptake by phytoplankton and in the freshwater literature in the early 2000s by Campbell et al. [53] and in 2004 by Hassler et al. [54], who questioned neglecting the contribution of the dynamic metal complexes to the metal uptake.

The key issue is the contribution of the metal complexes to the biouptake or the amount of metal reduced at the electrode. The dynamic nature of a complex is expressed by the interplay between the chemical dissociation kinetics and the diffusive transport (of the free metal and metal complexes) towards the interface, traversing a so-called diffusion layer generated due to the metal concentration gradient between the bulk and the uptaking surface.

The dynamic nature of the metal complexes is established by comparison of the time taken by the free metallic ion, M, to completely traverse said diffusion layer with the characteristic lifetime of the free metallic ion in the solution, which can be computed from the metal complex association (*k*_a_.c_L,t_) or dissociation (*k*_d_) rate constants and the total concentration of ligand L (c_L,t_). If the complexes are able to dissociate within the diffusion layer, they can contribute to the interfacial process that accumulates free metal ion and are called dynamic; otherwise, if they do not have time enough to dissociate, they cannot contribute to the metal flux and are called inert.

Dynamic complexes are further separated in labile or non-labile cases. Labile complexes are characterized by high rates of association/dissociation, meaning that complexation kinetics are much faster than the metal transport in the diffusion layer, thus the equilibrium between free metal and complexed metal is always maintained. In this case, the transport of the free and complex metal is coupled and must be described by an average diffusion coefficient. For quasi-labile or non-labile complexes, the magnitude of metal flux originating from complex dissociation approaches the diffusive metal flux, thus the overall surface flux will be a sum of diffusive transport and kinetic transport [55].

When applied to electrochemical stripping techniques, this means that the metal reduced in the deposition step is proportional to:

- the free metal in the bulk for inert complexes,
- the total metal in the bulk for the fully labile complexes
- the free metal plus a fraction of the metal complexes, for quasi-labile or non-labile complexes, depending on their degree of lability.

If the interpretation of the signal for these techniques is simple for both inert and fully labile complexes, it is quite complicated for the case when the complexes are not fully labile or inert. This led to the development of the first lability criteria by Davison in 1978 [56], using a thin mercury film electrode, and Van Leeuwen in 1979 [57], using the concept of reaction layer thickness developed by Koutecky and Koryta [58].

Even with these lability criteria, the experimental assessment of complex lability using voltammetric stripping techniques is quite difficult in general, becoming nearly impossible in the presence of polydisperse heterogeneous ligands, such as NOM. The main reason is that analytical descriptions of the voltammetric waves in ASV are impossible due to the complexity of the equations involved. Thus, numerical solutions must be used, in which case the dynamic parameters are not directly accessible.

This situation changed with the publication in 2004 [59] of an analytical wave equation for the kinetic current regime of the then-novel stripping technique Scanned Stripping Chronopotentiometry (SSCP), allowing direct experimental information on the dynamic nature of metal complexes. This started an intense period of linked theoretical and experimental development on chemodynamics of trace metal interaction with natural colloids and anthropogenic particles, spearheaded in 2005 by the publication by Pinheiro et al. [60], describing a novel theory for the trace metal dynamic speciation in colloidal dispersions.

The interested reader can follow this theoretical development in a series of reviews starting in 2009 with Van Leeuwen and Buffle [61], followed in 2012 by Mota et al. [62], and in 2017 by Van Leeuwen et al. [63]. Moreover, in 2017, Puy and Galceran [64] revisited the theoretical aspects of dynamic metal speciation with electrochemical techniques, focusing on the quantification of the contribution of complexes to the resulting flux via the lability degree. They concluded that there is a need for further developments regarding heterogeneous ligands and nanoparticles.

After these reviews, a few contributions of note have been made by Duval, Town and Van Leeuwen. In 2017 [65], these authors extended the reaction layer concept from small ligands to nanoparticulated complexing agents that contain metal binding sites at the surface or within their volume. They found that not excluding the particle volume from the reaction layer may overestimate the kinetic flux originating from dissociation of particulate metal complexes at the reactive surface. In 2018 [66], they presented an explicit mathematical expression for the lability of nanoparticulate metal complexes toward a reactive surface that depends on size, electrostatics properties of the particles, trace metal diffusion and dehydration rates pertaining to the loss of a water molecule in the metal ion inner hydration shell. The treatment applies for particles ranging from soft/core-shell to hard particles considering their volume and surface binding site distributions, respectively. Finally, in 2023 [67], they described the effect of the electric charge at a reactive interface on the diffusion rate of ionic species towards the interface and their local concentration profiles. They proposed a coupled steady-state Nernst-Planck equations for metal cations, ligands and metal complexes, including the correction for interfacial electrostatics and chemical kinetics, in order to obtain the metal surface flux and the spatial distributions of these species. Based on these equations, the complex lability was evaluated, and the authors found that the effect of interfacial electric field depends on the nature of the charges of the reactive species and surface, where, for example, repulsive interactions lead to a significant loss of lability.

### 2.2. Electroanalytical Field Measurements and Methodological Developments

The interest in trace metal speciation in natural waters can be traced to the publication of the book “Aquatic Chemistry” by Werner Stumm and J.J. Morgan in 1970 [68]. In the 1960s and 1970s, there was significant development of electroanalytical techniques due to the attribution of the 1959 Nobel Prize of Chemistry to Jaroslav Heyrovsky for the discovery of polarography, together with the introduction of electrochemical stripping techniques [69], and the advent of computer-controlled modern potentiostat/galvanostat instruments [70]. Therefore, it is not surprising that these new techniques were used at the trace metal speciation time to measure since they provided the lowest detection limits and affordable instrumentation.

An historical perspective of the evolution of the electroanalytical techniques for field surveys can be obtained by reviewing significant literature on the subject. In 1976, Nurnberg et al. [71] published a review on polarographic analysis of metals in aquatic systems, followed by comprehensive reviews in 1980 [72] and 1986 [73] by T. Mark Florence, where the authors already pointed out the predominance of anodic stripping voltammetry (ASV) to quantify trace element speciation in natural waters.

Despite its popularity for metal quantification or identification in aqueous systems, the ASV signal is difficult to interpret since the amount of metal reduced in the deposition step depends on the complexation dynamics in solution, and the signal measured in the reoxidation step is a current transient, characteristic of the voltammetric techniques applied in this stage (linear scan, differential pulse or square wave). Both the deposition and reoxidation steps are dynamic in nature; hence, relating the voltammetric signal with the trace metal speciation in solution is a mathematically involved problem.

In the 1980s and 1990s, literature reports were largely focused on signal interpretation difficulties for the deposition and reoxidation steps. One major issue was the discrimination of the metal species that contribute to the reactive transport of metal towards the electrode during the preconcentration step, which was already discussed in detail in the previous section. Another main difficulty in the interpretation of the ASV signal stems from the adsorption of organic matter at the mercury electrode surface [74,75,76,77], which has been shown to significantly affect the reoxidation signal such as Cd(II), Pb(II) and especially Cu(II) in ASV studies in natural samples [78].

Additionally, during the reoxidation step, voltammetric stripping techniques are also subject to surface metal concentration excess [79], meaning that the total metal concentration at the electrode surface will be significantly higher than the bulk metal concentration and possibly of the same order of magnitude or larger than the total ligand concentration, provoking peak distortions. In combination with the possible organic adsorption at the electrode surface, these effects are amplified so that in extreme cases, they may even lead to the formation of peak shoulders [80], rendering the exploitation of peak height impossible for quantification purposes.

Numerous strategies have been developed for overcoming interference from adsorption, the most common of which involves some modification of the electrode surface, typically the application of a polymer coating (e.g., Nafion^®^) [81], polystyrene sulphonate PSS [82] or Nafion/PSS mixtures [83]). Amongst others approaches, several works have proposed, for example, a medium exchange procedure where the deposition is carried out in the sample solution and the reoxidation in a pure electrolyte solution [84], an acidification step where the deposition is achieved at the sample pH and reoxidation at pH < 2 [85], or a coating of the electrode with a gel layer as suggested by Tercier and Buffle in 1996 [86]. This latter approach evolved by various steps culminating in the Gel Integrated Microelectrode array (GIME) [17,87], where a 150 µm thick agarose gel layer protects the Hg-plated Ir microelectrodes array from fouling. Moreover, pseudo-polarography, which is constructed by plotting ASV stripping peak current versus deposition potential, has been applied to freshwater systems to determine the stability constants of labile metal complexes, e.g., for Zn element in Vega in 1995 [88] and later in 2006 for Cd species in Tsang et al. [89].

To carry out field electrochemical stripping measurement, another challenging task is to reduce the dissolved oxygen interference that commonly distorts electrochemical stripping techniques signals. In the laboratory, the easier option is to purge the solution with Nitrogen or Argon, while for field speciation studies, this procedure cannot be applied since degassing removes CO_2_ from solution, hence changing the pH and unbalancing the cation speciation in solution. To address this issue, Tercier and Buffle proposed an online oxygen removal system based on the permeation of oxygen through a silicone tubing surrounded by an enzymatic cross-linked O_2_ scavenging gel [90].

Both the GIME and the proposed online oxygen removal were used in the development of the most successful ASV field probe named the “Voltammetric in situ profiling system” (VIP), which has been developed by Geneva University and commercialized by Idronaut for more than two decades [17].

**Figure 1:**
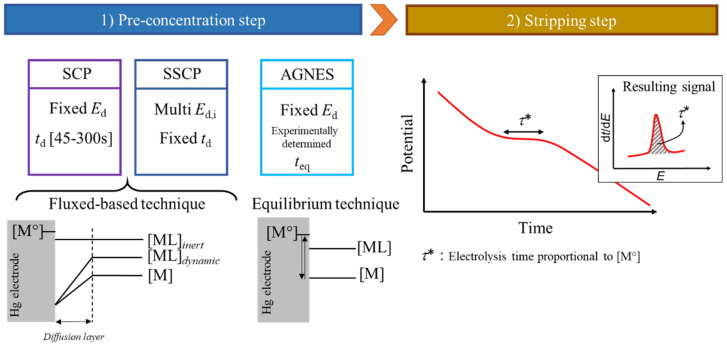
Generic description of SCP, SSCP and AGNES.

In the first half of the 2000s, three novel stripping techniques were introduced that significantly changed the panorama of electrochemical trace metal speciation. In 2001 and 2002, Town and Van Leeuwen introduced SCP [91] and SSCP [92], respectively, and in 2004, Galceran et al. developed the equilibrium technique of Absence of Gradients and Nernstian Equilibrium Stripping (AGNES) [93]. The principal characteristics of these techniques are presented in Figure 1, where it is shown that the pre-concentration steps are all different, while the stripping step is identical.

SCP is similar to Jagner’s Potentiometric Stripping Analysis (PSA) [94] but applies a much lower reoxidation current. In such a way, at the end of the stripping step, all the metal previously reduced is reoxidised, namely full depletion of the metal deposited. During the second stage, SCP measures the variation of electrode potential (*E*) with reoxidation time (*t*) that reflects the metal electrolysis from the electrode to the solution. Then, the amount of deposited metal during the preconcentration step can be computed using the Faraday law, resulting in a direct proportion between the limiting transition time (τ*) and the metal concentration in the sample [91]. In SCP, the limiting transition time is obtained from the peak area of the plot d*t*/d*E* vs. *E*, with good discrimination regarding the capacitive charge that appears under the peak baseline and avoiding the problems of signal distortion in the voltammetric stripping techniques caused by adsorption or surface excess. Thus, SCP offers an excellent detection limit comparable to differential pulse (DP)-ASV or square-wave(SW)-ASV, is not affected by organic matter adsorption [95], and is less sensitive to both the surface excess problems and the formation of intermetallic complexes in the amalgam [96]. However, unlike other voltammetric techniques, the SCP wave potential or waveform does not provide any useful thermodynamic information [97]. To overcome this limitation, a new technique (SSCP) was proposed to gain access to potential and waveform information while keeping the aforementioned SCP advantages. An SSCP wave is constructed from a series of individual SCP measurements obtained at different deposition potentials (*E*_d_), ranging from the situation where no metal ions are reduced, passing by the Nernstian region, and ending in the diffusion-limited limit where all metal ions arriving at the electrode are reduced [92].

SSCP is actually the most complete technique for trace metal speciation analysis, essentially due to the analytical equation that describes the SSCP wave in the kinetic regime [59]. By comparing the SSCP curves in the presence and absence of ligands (generally called SSCP calibration), the decrease in limiting transition time will provide information on the diffusion coefficient and association rate constant of the metallic complexes, while the shift in half-wave deposition potential will provide the thermodynamic stability constant of the complexes, independent of the dynamic nature of said complexes (using an expression analogous to DeFord-Hume’s for a voltametric wave) [98]. The thermodynamic stability constant can also be computed from the variation of limiting transition time in the presence and absence of complexes as long as the metal complexes are fully labile and their diffusion coefficient (*D*_ML_) is known [99]. Thus, the SSCP waves contain an integrated lability criterion quantitively accessible in the case of metal binding by chemically and physically homogeneous ligands, by simple comparison of the experimental decrease in transition time in the presence of ligands and the predicted decrease based on the thermodynamic stability constant obtained from the half-wave potential shift [100].

The presence of heterogeneous ligands affects the slope of the SSCP wave due to the variation of the stability constant with the metal-to-ligand ratio effective at the electrode surface. This feature can be exploited to obtain partially the complexation isotherm and the degree of heterogeneity [101], however, the experimental lability diagnosis is no longer quantitative, being only able to provide a tendency to gain or lose lability as a function of different metal-to-ligand ratios in the bulk.

The most important interference in SCP/SSCP is the dissolved oxygen since it can chemically reoxidize the metal during the stripping step and thus decreasing the signal. This oxygen competition is especially important for mercury drop electrodes where the reoxidation currents must be very small, of the order tens of nanoamperes, to assure full metal depletion. Hence the best electrodes to carry out SCP/SSCP experiments are thin mercury film electrodes (TMFE) [102] due to their low area/volume ratio and much higher reoxidation currents, of the order of microampere, leading to much faster experiments and in turn lower oxygen interference [103]. SCP/SSCP can also be implemented in solid electrodes, such as bismuth [104,105] or gold electrodes [106], facilitating their utilization in field studies where the use of mercury electrodes may pose problems.

AGNES [93] is an equilibrium technique that is also based on a stripping stage during the reoxidation process and can be operated in connection with SCP/SSCP [107]. This method is used to determine directly the free metal concentration in solution, and consequently, the equilibrium stability constant of the metal complexes. The first step in AGNES is very different from all the other stripping techniques since two conditions need to be achieved: (1) the potential applied to the electrode (*E*_1_) lies within the Nernstian region for the metal ion under study and (2) the deposition time must be long enough so that equilibrium between the reduced metal at the electrode and the free metal in solution is attained.

The reoxidation step is only used to quantify the amount of reduced metal ions during the deposition step. Different electrochemical techniques were tested for this purpose before choosing a chronopotentiometric reoxidation procedure [108]. The free metal ion in solution can be then computed from the reduced metal at the electrode using Nernst equation, independent of other solution parameters such as metal complexation or organic adsorption at the electrode [109].

The TMFE is the optimal platform for performing AGNES due to its fast equilibration time promoted by the large electrode area for a very small electrode volume, hence yielding significantly shorter experimental times as compared with mercury drop electrodes [110]. Remarkably AGNES can also be carried out in solid electrodes, as long as the amount of metal reduced is low enough to prevent full coverage (monolayer) of the electrode surface, as demonstrated for the determination of lead on a bismuth electrode [111] and copper in a gold electrode [112].

To complete the information on trace metal speciation in natural systems, we suggest reading the following three reviews. In 2006, Sigg et al. [113] published a study of several analytical techniques for dynamic trace metal speciation in natural waters. Five metals (Cu, Zn, Cd, Pb, Ni) were tested by various passive sampling techniques: DGT; flow-through and hollow fiber PLM and DMT, and three voltammetric techniques: gel-integrated microelectrodes combined with a voltammetric in situ profiling system (GIME-VIP); SCP and CLE-CSV.

Pesavento et al. [114] presented in 2009 a review on the analytical methods for determination of free metal ion concentration, labile species fraction, and metal complexation capacity of environmental waters. The review describes electrochemical, including ASV, CLE-SV, SCP, AGNES and voltammetric in situ probes, and non-electrochemical techniques covering separation techniques based on ion exchange (IE) complexing resins (CR), and micro-separation methods such as the DMT, DGT and PLM.

Companys et al. [115] reviewed in 2017 the performance of the techniques CLE-SV, SCP/SSCP and AGNES for trace metal speciation in diverse environmental media (seawaters, freshwaters and soil extracts), including their working principles and an evaluation of strong and weak points.

### 2.3. Critical Review of the Last Five Years

Regarding in situ electroanalytical measurements, the VIP/TracMetal sensing probes are a notable achievement and represent the most advanced devices, being the only ones that are commercially available. They are mostly used in seawater or estuaries due to the larger community of electrochemical marine scientists, but there is nothing preventing their use in freshwaters. In recent years, Illuminati et al. (2019) [116] reported the use of five VIP systems and one Multi Physical Chemical Profiler, as well as conventional voltammetric instruments, during a cruise along the Po River plume to evaluate the distribution of Cd(II), Pb(II), and Cu(II) between different fractions (free ion, dynamic, colloidal, dissolved and particulate). The evolution of the fractions was assessed in the water column during estuarine mixing. It was observed that all the metal fractions decreased following the salinity gradient, and only the Cu dynamic fraction was likely to be toxic to the phytoplankton.

Tercier-Waeber et al. presented in 2020 the development and field validation of newly designed nanostructured gold-plated gel-integrated microelectrode arrays (Au-GIME) applied using in situ Square Wave Anodic Stripping Voltammetry (SW-ASV) to evaluate the potential bioavailable inorganic mercury (Hg(II)) species in coastal areas [117] and inorganic arsenite (As(III)) elements in fresh and marine aquatic systems [118]. The Au-GIME consists of arrays of one hundred to five hundred interconnected iridium (Ir)-based microdisks electroplated with renewable Au nanoparticles (AuNP) or Au nanofilaments (AuNF) and covered with an agarose gel. The AuNF-GIMEs have detection and quantification limits at the low pM level for Hg(II) and sub-nanomolar for As(III), thus fulfilling the requirement for their direct monitoring.

Following the development of the AuNF, Tercier-Waeber et al. [8] reported in 2021 the development of a submersible multichannel trace metal sensing probe (TracMetal). This probe allows in situ measurements of the dynamic fraction of Hg(II), As(III), Cd(II), Pb(II), Cu(II), Zn(II). It incorporates nanostructured Au-plated and Hg-plated GIMEs for which the authors stated sensitivities in the range of pM levels for Hg(II) and sub-nM for the other metals with precision lower than 12%. The TracMetal is capable of autonomous operation, data storage and wireless data transfer. The system was successfully applied in the Arcachon Bay (France) to study the temporal variation of the dynamic fraction of the targeted trace metals.

Layglon et al. [119] reported in 2022 the dynamic fraction concentrations of Cu(II), Cd(II), Pb(II) and Zn(II) in the Genoa harbor waters (Italy), obtained by SWASV on a GIME (TracMetal sensing probe). Trace metals in the operationally defined dissolved <0.2 μm and <0.02 μm fractions were equally quantified through sampling/laboratory-based techniques. The obtained results showed a clear spatial trend for all studied metals, from the enclosed contaminated part of the harbor towards the open part. The same methodology and TracMetal probe were also employed by Abdou et al. in the Gironde Estuary (France), first for studying the speciation and partitioning of Pb(II), Cd(II) and Cu(II) along the salinity gradient (S = 0.10 to S = 34.0) [120], and then for examining the impact of phytoplankton activity on trace metal speciation and partitioning at a short temporal scale [121]. In this study, the voltammetry quantification of dynamic trace metal fraction potentially bioavailable was carried hourly in situ, together with a complementary surface water sampling for the quantification of the targeted trace metals in the dissolved fractions, in suspended particles, and in the phytoplankton nets. The relevant acquisition frequency offers the possibility to monitor in real-time, for example, the dynamic Cd(II) and Cu(II) regeneration related to algal cells under post-bloom conditions.

The VIP/TracMetal provides nice analytical performances in seawater or estuarine conditions due to the high salinity, constant pHs and small NOM concentrations (0.5–5 mg/L). However, in freshwaters where the NOM concentrations are normally higher (5–50 mg/L), the colloidal size distribution is broader, and both salinity and pH can vary significantly. Therefore, it will be more difficult to estimate the contribution of free and complexed metal ligand in the electrochemical signal. Effectively, both the free-metal ions and smaller metal complexes will permeate the gel with diffusion coefficients equal or closer to those observed in solution, while larger colloidal or nanoparticulated complexes will either be excluded or will move through the gel with unknown diffusion coefficients, rendering the electrochemical signal interpretation more intricate.

In addition to the VIP system, a few autonomous devices have been described in the literature in the last five years, mostly dedicated to seawater. Hence, we provide here the example of two devices that can also be used in freshwaters. Lv et al. described in 2017 [122] a fully automated electrochemical measuring equipment. It consisted of a simplified electrochemical instrument capable of carrying out cyclic voltammetry (CV), linear sweep voltammetry (LSV), differential pulse voltammetry (DPV), and square wave voltammetry (SWV), coupled with an automated flow-injection system and network communication technology. Online monitoring using a mercury film plated on a carbon electrode was tested in a waterwork, and the accuracy of the instrument was confirmed by comparison with standard analytical methods, albeit the system is limited since it measures in an acetate buffer. In 2022, De Vito-Francesco et al. [123] proposed a water surface vehicle capable of semi-autonomous driving and equipped with a microfluidic device containing a potentiostat for on-site quantification of Pb(II) and Cu(II) in surface waters. The detection is based on the method of SWASV using carbon-based screen-printed electrodes (SPE). The system presented a bias of 75% for Pb(II) and 65% for Cu(II), a relative standard deviation of 11–18% for Pb and 6–10% for Cu, and a limit of detection of 19 nM for Pb(II) and 110 nM for Cu(II), which are not low enough for most natural water measurements The lifespan of a SPE averaged 39 measurements per day. Albeit technically excellent outcomes, the cited work neglects to address some of the major analytical problems of measuring trace metal ions in natural water, such as the ubiquitous adsorption of organic matter at the electrode surface.

As for the field application of the new techniques (SCP, AGNES and SSCP), Hackel et al. [124] presented in 2021 a fast screening method for field determination of total Pb(II) and Zn(II) using Cd(II) as internal standard. This work was carried by SCP on acidified sample solutions using TMF-SPEs. The authors reported very low detection limits of 0.06 nM for Pb(II) and 0.04 nM for Cd(II) using the standard addition method with good agreement with the results obtained by inductively coupled plasma mass spectrometry (ICP-MS). Oxygen removal is critical for SCP/SSCP; thus, reliable field measurement using these techniques must have an efficient way of carrying out this task. Rotureau et al. [125] investigated in 2021 an oxygen elimination system where dissolved oxygen was removed locally in the vicinity of a sensor by the means of electrochemical oxygen filter constructed from platinum grids [126]. The electrochemical device was tested for Cd(II), Pb(II) and Zn(II) determination in electrolyte solutions and in a natural water sample, using SCP at a thin mercury film screen-printed electrode. The oxygen filter showed a good level of performance, removing the need to purge the sample, and the device provided limits of detection of metals in the nanomolar range. In 2022, Rosales-Segovia et al. [127] presented a methodology to study Zn(II) species in natural waters based on four speciation techniques: AGNES, polymer inclusion membranes (PIM), linear scan ASV and DGT. The technique was tested in a Catalan river (Spain) where AGNES and PIM provided the free metal in the sample, while ASV and DGT measured two different “labile” fractions, since they are related to the characteristic diffusion layer scale pertaining to each technique, i.e., the diffusion layer for the electrode sensor or the gel layer thickness for the DGT device. The authors claim that the “combination of the information retrieved by the techniques allowed to quantify lability degrees of the pool of Zn(II) complexes and to build up the effective concentration signature for this water”. This work displays a very interesting coupling of complementary approaches for metal speciation studies, but data acquisition is very time-consuming, yielding scarce experimental data in final. It would be necessary to extend this type of field work to a broader range of natural samples to fully evaluate its robustness.

Chen et al. [128] studied the influence of dissolved organic matter (DOM) properties on the speciation of Pb(II), Zn(II), and Cd(II). For that purpose, the authors used AGNES to construct metal binding isotherms in the presence of allochthonous or autochthonous DOM and compared the results with the ones predicted by WHAM. They observed that under the same conditions (physicochemical composition of the medium), the allochthonous DOM showed a higher level of metal binding than the autochthonous DOM, following the order Pb(II) > Zn(II) > Cd(II). The authors stated that “WHAM performance was affected by source variation through the active DOM fraction”, namely the effective DOM portion participating in metal binding.

An important methodological advance was reported in 2020 by Pinheiro et al. [129], where they demonstrated that the SSCP wave can be converted to binding curves a low metal to surface coverage for labile heterogeneous macromolecular systems, provided that SSCP is used in conjunction with AGNES to determine independently the free metal ion in the bulk solution. Effectively, the different deposition potentials applied at the SSCP wave investigate different values of the free metal and complex concentrations at the electrode surface, giving access, via a mathematical treatment, to a window of the metal binding curve with a chemically heterogeneous ligand. Examples with polystyrene sulfonate, Laurentian Fulvic acid and a peat humic acid were reported. Still in 2020, Rotureau et al. [130] adopted this methodology to investigate the binding heterogeneity of Cd(II), Pb(II) and Zn(II) with silica nanoparticles (7.5 and 17 nm radius) for a range of pHs and ionic strengths. As discussed in this paper, analyzing the degree of heterogeneity according to the type of metals or the nature of particles can reveal useful information on the structural or chemical features of the reactive colloidal interphase.

In addition to the traditional metals of environmental interest (Cd(II), Cu(II), Pb(II), Zn(II)), there is growing interest in the environmental fate of so-called strategic metals due to their growing use in electronic devices and batteries. One such metal is Indium, which has received considerable attention recently. In 2018, Tehrani et al. [131] proposed the determination of In(III) using AGNES in the hanging mercury drop electrode (HMDE), reporting sub-nanomolar detection limits, and investigated indium binding with nitrilotriacetic acid and oxalic acid. The following year, the same group proposed a new analytical methodology to provide guidelines to AGNES deposition times and investigated In(OH)_3_ solubility product [132]. In 2019, Rotureau et al. [133] reported the quantification of total and free indium concentrations in solution using SCP and AGNES on a thin mercury film/rotating disk electrode (TMF/RDE). They obtained limits of detection of 0.5 nM for SCP and 0.1 nM for AGNES that can be further improved using longer experiment times. In 2022, Rotureau et al. [134] presented a study on the intrinsic stability of indium with humic matter, where the experiments were carried out in NaClO_4_ electrolyte (10–100 mM, pH 4) at different metal-to-humics concentration ratios using AGNES. The obtained results evidence an important impact of electrostatics on indium complexation by humic matter.

AGNES has also been extended to another metal element, the Antimony, by Pla-Vilanova et al. [135]. Due to the extensive hydrolysis of Sb(III), the calibrations and AGNES measurements were carried out for the aqueous Sb(OH)_3_ concentration instead of the free antimony. The authors validated their method by comparing their experimental values of Sb(OH)_3_ titration with oxalate with the values predicted with the complexation constants from the literature.

Electroanalytic applications for the aqueous speciation studies of rare earth elements are scarce. Nevertheless, a recent work on the free Eu(III) determination by the coupling between electroanalytical techniques and DMT device has been proposed by Janot et al. [136]. In this work, Eu(III) concentrations were quantified in the acceptor solution of the DMT by applying CLE-SV using *N*-nitroso-*N*-phenylhydroxylamine (cupferron) as the adsorbing ligand. The total Eu(III) concentration in the acceptor can then be related to free Eu(III) in the donor, i.e., the natural sample. However, free Eu(III) tends to adsorb strongly to the cation-exchange membrane; thus, at least 100 mM of Ca(II) was necessary to make adsorption of Eu(III) onto the membrane negligible, which may change the speciation in certain natural samples.

To complete this overview, we suggest to the readers a few tutorial reviews to help become more familiar with the use of these speciation techniques. In 2019, Borril et al. [137] discussed the experimental use of ASV, including the choice of electrode material and solution composition (pH, electrolyte, buffer). The pH dependence of metal speciation and possible intermetallic effects were also pointed out. The authors considered the differences between measuring model solutions and environmental samples containing organic matter, biological and inorganic species, which can themselves adsorb metal ions. In addition to that, in 2019, Town and Van Leeuwen reviewed SSCP [138] and compared this analytical approach with voltammetric and other dynamic speciation techniques. The authors highlighted that the “complete depletion regime of SSCP offers particular advantages due to its ability to distinguish between various factors that can confound interpretation of data from other electroanalytical methods, including electrochemical irreversibility, kinetically controlled currents, reduced diffusion coefficient of the metal complex species as compared to the free metal ion, and chemical heterogeneity in the intrinsic binding affinity”. In 2022, Lopez-Solis et al. [139] addressed the fundamental concepts of AGNES and provided the experimental steps to implement it, including ready-to-run files for the software controlling the potentiostat, computation spreadsheets, step-by-step laboratory protocols, together with two practical case studies for determining free Zn(II) concentration.

## 3. Road Map

In our point of view, the combined utilization of SCP, AGNES and SSCP during a field campaign may significantly contribute to a complete trace metal cation speciation analysis, including the quantification of total metal by SCP, free hydrated cation by AGNES and insight into the heterogeneity and dynamic nature of metal complexes by SSCP.

Sample characterization by independent techniques and size separation steps may be necessary to identify the different complexed and adsorbed species (mineral particles, micro-organisms). In this section, we will propose a road map for the development of trace metal cation speciation based on these concepts.

### 3.1. Total Metal Ion Screening

Trace metal cation concentrations in freshwaters vary from the natural abundance of the metal to various degree of pollution. In non-polluted samples, some metal ions are commonly found well above the detection limits of the techniques (Cu(II), Zn(II)), while others generally lie close these limits (Pb(II)) and others below (e.g., Cd(II), In(III)). In pristine freshwater, trace metal ion concentrations are relatively stable over large periods of time, while in polluted samples, the values are higher or much higher than the “normal” value but may vary dramatically in short periods of time (~days) depending on the nature of the contamination (spike or continuous) or natural seasonal events (flood or low-water periods) and the associated processes such as contaminated sediment remobilization.

It is pointless to carry out speciation measurements for metal ions whose total concentrations in the sample are close or below the detection limit of the analytical techniques. Thus, a fast total metal screening technique such as the one proposed by Hackel et al. [124] should be used to sort out the samples to be put forward to full dynamic speciation analysis on those where the total metal concentration is too low to be considered.

### 3.2. Dynamic Speciation Methodology

**Figure 2:**
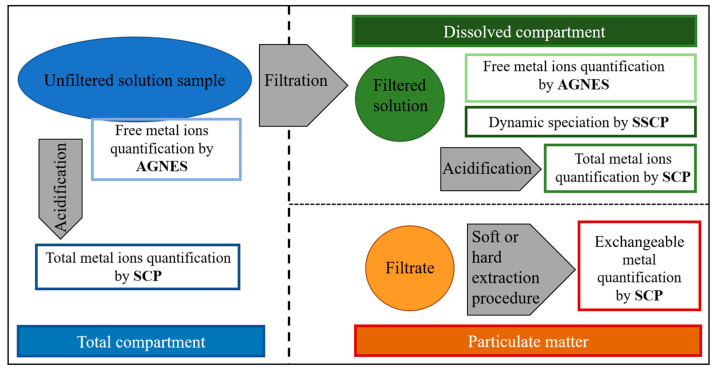
Scheme of the proposed dynamic speciation methodology.

The proposed dynamic speciation methodology is presented in Figure 2 and is divided into four steps. The first step consists of the determination of the total and free metal cations measured in the pristine sample.

Then the sample is filtered through a 0.45 or 0.22 µm membrane. The filtered solution contains the so-called dissolved ligand fraction, and the particulate ligand fraction is the one retained in the filter.

From the filtered solution, two aliquots are taken, and the first is acidified to a pH below two, being the dissolved total metal concentration that could be measured by SCP, while in the second, the free metal concentration in the dissolved compartment could be quantified by AGNES. In the third step, as long as the total dissolved metal concentration is sufficiently above the SCP detection limit, an SSCP measurement will be carried out in the filtered sample to gain information on the heterogeneity and dynamic nature of the dissolved complexes.

Finally, the fourth step consists of extracting the metal from the particles retained on the filter by means of an acid washing, followed by SCP quantification.

At the end of this speciation methodology, we should be able to take stock of the quantitative metal speciation by determining the total metal ions in the pristine sample, the dissolved phase and retained in the particles, the free metal in the pristine solution and dissolved phase, and the SSCP information on heterogeneity and dynamic nature of the complexes of the dissolved phase.

Just recalling here that carrying out SSCP in the pristine solution (or unfiltered solution sample) is not relevant since the larger particles and organic colloids retained in the filter will not contribute for the dynamic SSCP due to their low diffusion coefficients, as discussed in the theoretical background.

### 3.3. Additional Information Necessary for the Sample Characterization

During the field measurement, complementary parameters are needed to better understand the chemical status of metallic elements: first, pH, temperature and conductivity should be measured on-site and *always kept constant during the trace metal cation quantification*. The method calibrations should be carried out at the same conductivity to minimize errors.

Since the NOM is the principal complexing agent for the metal ion, it is required to quantify the total organic carbon (TOC), dissolved organic carbon (DOC) and particulate organic carbon (POC). In case of sufficiently high NOM concentrations, it will be helpful to carry out a proton titration between pH 3 and 10.5 to obtain direct information on the carboxylic and phenolic groups abundance.

Regarding the particulate fraction retained in the filter, first, it will be necessary to quantify it by weight, followed if possible by an identification of the mineral phases present, to establish the main solid components (clays, iron hydro-oxides, calcium minerals).

Finally, it would be helpful to quantify the major cations and anions for a better sample description, especially for estimating the Ca(II) and Mg(II) competition with the trace metal cations.

## 4. Future Work Recommendations

Regarding the interpretation of speciation measurements of freshwaters, one of the major questions remaining is the influence NOM adsorption at the surface of natural particles. At this point, the few experimental studies that have been carried out focus on the metal binding to humic matter adsorbed on iron oxides, which is described using an independent combination of NICA-Donnan and CD-MUSIC models [140]. More experimental work is clearly needed to address the metal binding properties of particles such as clays and calcium-containing entities covered by NOM instead of well-purified organic materials. Moreover, additional theoretical development is necessary to understand the variation of both proton and metal binding parameters with NOM-coated particles as compared with the isolated NOM and uncoated particles.

One of the keys of development will be the ability to provide affordable automatic instruments to carry out metal speciation in the field, allowing the deployment of multiple setups during a field survey, thus significantly increasing the number of experimental measures either in frequency or in geographical area coverage. Only the availability of large datasets will allow determining and refining the parametrization of predictive models that represent the fundamentals for environmental risk assessment studies.

Finally, it will be important to extend the proposed approaches to a larger variety of metals cations. At this point, AGNES and SSCP methodologies are available for Cd(II), Pb(II), Zn(II) and In(III) in mercury electrodes, Pb(II) in bismuth electrodes and Cu(II) in gold electrodes. Albeit these metal cations are amongst the most important toxic species in freshwaters, it will be important to also be able to measure, for instance, Hg(II) and Ag(I). There are several possible development pathways, one of the most promising being the exploitation of under-deposition potential in solid electrodes, as carried out for the Pb(II) in bismuth electrodes [111].

The unique abilities of electroanalytical stripping techniques to perform trace metal cation dynamic speciation provide the best way to investigate in-depth and integrative analysis directly in the field. For this reason, further challenging research is still necessary to develop both the instrumentation and the theory aspects pertaining to these electrochemical methods with the aim of better understanding the metal fate and reactive transfer in environmental systems.

## Data Availability

Not applicable.
